# Host Genetic Variation Impacts SARS-CoV-2 Vaccination Response in the Diversity Outbred Mouse Population

**DOI:** 10.3390/vaccines12010103

**Published:** 2024-01-20

**Authors:** Marta C. Cruz Cisneros, Elizabeth J. Anderson, Brea K. Hampton, Breantié Parotti, Sanjay Sarkar, Sharon Taft-Benz, Timothy A. Bell, Matthew Blanchard, Jacob A. Dillard, Kenneth H. Dinnon, Pablo Hock, Sarah R. Leist, Emily A. Madden, Ginger D. Shaw, Ande West, Ralph S. Baric, Victoria K. Baxter, Fernando Pardo-Manuel de Villena, Mark T. Heise, Martin T. Ferris

**Affiliations:** 1 Genetics and Molecular Biology Curriculum, University of North Carolina, Chapel Hill, NC 27599, USA; mcruzcis@email.unc.edu (M.C.C.C.); bh2907@cumc.columbia.edu (B.K.H.); 2 Department of Genetics, University of North Carolina, Chapel Hill, NC 27599, USA; breantie_parotti@med.unc.edu (B.P.); ssarkar@southernresearch.org (S.S.); sharon_taft-benz@med.unc.edu (S.T.-B.); timothy_a_bell@med.unc.edu (T.A.B.); matthew_blanchard@med.unc.edu (M.B.); phock@email.unc.edu (P.H.); ginger_shaw@med.unc.edu (G.D.S.); fernando_pardo-manuel@med.unc.edu (F.P.-M.d.V.); mark_heisem@med.unc.edu (M.T.H.); 3 Division of Comparative Medicine, University of North Carolina, Chapel Hill, NC 27599, USA; lizand@email.unc.edu (E.J.A.); vbaxter@txbiomed.org (V.K.B.); 4 Department of Microbiology and Immunology, University of North Carolina, Chapel Hill, NC 27599, USA; jacob_dillard@med.unc.edu (J.A.D.); emily_madden@outlook.com (E.A.M.); rbaric@email.unc.edu (R.S.B.); 5 Department of Epidemiology, University of North Carolina at Chapel Hill, Chapel Hill, NC 27599, USA; leist@email.unc.edu (S.R.L.);; 6 Department of Pathology and Laboratory Medicine, University of North Carolina, Chapel Hill, NC 27599, USA; 7 Texas Biomedical Research Institute, San Antonio, TX 78227, USA; 8 Lineberger Comprehensive Cancer Center, University of North Carolina, Chapel Hill, NC 27599, USA

**Keywords:** Diversity Outbred, host genetic diversity, SARS-CoV-2, vaccination

## Abstract

The COVID-19 pandemic led to the rapid and worldwide development of highly effective vaccines against SARS-CoV-2. However, there is significant individual-to-individual variation in vaccine efficacy due to factors including viral variants, host age, immune status, environmental and host genetic factors. Understanding those determinants driving this variation may inform the development of more broadly protective vaccine strategies. While host genetic factors are known to impact vaccine efficacy for respiratory pathogens such as influenza and tuberculosis, the impact of host genetic variation on vaccine efficacy against COVID-19 is not well understood. To model the impact of host genetic variation on SARS-CoV-2 vaccine efficacy, while controlling for the impact of non-genetic factors, we used the Diversity Outbred (DO) mouse model. We found that DO mice immunized against SARS-CoV-2 exhibited high levels of variation in vaccine-induced neutralizing antibody responses. While the majority of the vaccinated mice were protected from virus-induced disease, similar to human populations, we observed vaccine breakthrough in a subset of mice. Importantly, we found that this variation in neutralizing antibody, virus-induced disease, and viral titer is heritable, indicating that the DO serves as a useful model system for studying the contribution of genetic variation of both vaccines and disease outcomes.

## 1. Introduction

The emergence of SARS-CoV-2 has caused nearly 7 million deaths globally, including more than 1 million deaths in the United States, and it is estimated that the global pandemic will cost the world economy over 12 trillion dollars by 2024 [1,2]. Due to the extraordinary public health threat posed by SARS-CoV-2, there has been an unprecedented effort devoted to the development, testing, and deployment of a variety of SARS-CoV-2 vaccines. These vaccine platforms, which include mRNA, vectored, recombinant protein, virus like particles (VLPs), and inactivated whole virus (iCoV) vaccines, have been proven to safely elicit protective immunity against SARS-CoV-2 [3,4,5,6]. However, even within a given vaccine modality, there is significant variation in SARS-CoV-2 vaccine efficacy across the population. This variation has been driven in part by the rapid emergence of viral variants of concern (VOC), but also individual-to-individual variation in vaccine response [7,8]. Understanding the factors that influence this individual-to-individual variation in vaccine performance has important implications both for ongoing efforts to control SARS-CoV-2, as well as other disease threats such as influenza A virus or other future pandemic threats.

Prior research has shown that factors such as advanced age, compromised immune function, or prior antigen exposure can affect an individual’s ability to mount protective vaccine-induced immune responses [9,10,11]. In addition to these factors, there is a growing body of evidence suggesting that host genetic variation can influence an individual’s response to vaccination. The efficacy of several vaccines, including vaccines against influenza virus, the measles mumps and rubella vaccine (MMR), as well as the hepatitis B vaccine has been shown to be impacted by polymorphic host genes [12,13,14,15,16]. More recently, some targeted associations between SARS-CoV-2 susceptibility alleles and vaccine responses have been shown in a cohort of human vaccinees from Italy, where various polymorphisms were identified as affecting IgG levels and neutralizing antibody response [17]. However, our understanding of how variation in host genes affects SARS-CoV-2 vaccine-induced immunity is still incomplete.

Given the multitude of environmental, genetic, and demographic factors that can impact vaccine efficacy in humans, it can be difficult to deconvolute their influence on vaccine outcomes in human studies. Mouse models have been a crucial model system for the preclinical evaluation of SARS-CoV-2 vaccines as they allow investigators to control a variety of potentially confounding factors, including diet, prior pathogen exposure, or genetics [18,19]. However, this experimental strength can also be thought of as a limitation, as any given inbred mouse strain (e.g., BALB/c) does not recapitulate the genetic diversity observed in outbred human populations. These strains are limited in their utility for analyzing the impact of host genetic variation on vaccine performance or other aspects of host immunity [20,21,22]. There are several experimental mouse models which incorporate characterizable genetic variation, which can be used to assess host genetic effects while controlling other factors. One of these, the Diversity Outbred (DO) mouse, is an outbred mouse population derived from eight founder strains, composed of five traditional laboratory strains (A/J, C57BL/6J, 129/S1/SvImJ, NOD/ShiLtJ, NZO/HILtJ) and three inbred, wild-derived strains (PWK/PhJ, CAST/EiJ, and WSB/EiJ) [23]. While the DO is derived from the same founder strains as the Collaborative Cross (CC), each mouse is genetically unique and heterozygous at the majority of loci, better representing outbred genetics observed in human populations [24]. The genetic diversity within the DO population has already proven useful for studying the role of host genetics in infection and vaccine responses against pathogens such as tuberculosis and influenza [25,26,27,28]. Therefore, the DO serves a relevant model that can recapitulate the heterogeneity of vaccine and disease phenotypes in a human population and determine the role of host genetics.

To assess the impact of host genetic variation on vaccine induced immunity, we designed a case–control study with a population of DO mice. Mice were immunized with an S2P protein subunit vaccine in combination with the experimental RIBI adjuvant or mock vaccinated. Following vaccination, DO mice were challenged with a mouse-adapted MA10 strain of SARS-CoV-2. We found that the development of neutralizing antibodies had a strong genetic component, consistent with human studies [17]. Furthermore, we found that while vaccination protected most mice from SARS-CoV-2 challenge, a subset of vaccinated mice remained susceptible SARS-CoV-2-induced disease. We found that where there was vaccine failure in mice, viral titers remained high despite the presence of virus neutralizing antibodies. We also found viral titers present within a subset of mice that did not experience weight loss. These results indicate that host genetic diversity can impact vaccine efficacy and indicated that the DO and related mouse genetic reference populations are useful resources for studying how host genetic polymorphisms impact vaccine efficacy.

## 2. Materials and Methods

### 2.1. Virus Stocks

Mouse-adapted SARS-CoV-2 MA10 was prepared as previously described [29,30].

### 2.2. Mice

The mouse studies were performed in strict accordance with the recommendations in the Guide for the Care and Use of Laboratory Animals of the National Institutes of Health. All of the mouse studies were performed at UNC using protocols approved by the UNC Institutional Animal Care and Use Committee (IACUC). Male Diversity Outbred mice were obtained from Jackson Laboratory (Cat: 009376) at 4–5 weeks of age, and tail-clipped for genotyping. Males were singly housed for 10 months prior to immunization. The mice were kept on standard mouse chow, with ad libitum access to water, and a 12:12 light: dark cycle.

### 2.3. Genotyping

Whole genomic DNA from tail snips was extracted using QiaGen DNEasy Blood and Tissue kits. We sent 1.5 µg of DNA per sample, in a minimum volume of 20 µL in 96-well plates, to Neogen (Lincoln, NE, USA) for genotyping on the MiniMUGA array [31].

### 2.4. Genetic Clustering

We took our MiniMUGA genotypes, and filtered down from the 10,656 genomic markers present on the autosomes and X-chromosome to only those that were informative amongst the founders of the DO population [31]. We further filtered these markers to a set of 874, which were diagnostic for a given founder in the context of the DO (Supplementary Materials, Data S1 and Data S2). We then utilized this marker set to determine the relative relatedness between each pair of DO mice in our population.

For each pair of animals, we assessed relatedness by scoring their sharing of minor alleles. Pairs of mice were scored as follows at each marker:

Major allele homozygous/major allele homozygous = 0;

Major allele homozygous/heterozygous = 0;

Major allele homozygous/minor allele homozygous = 0;

Heterozygous/heterozygous t = 1.2;

Het/Minor allele homozygous = 1;

Minor allele homozygous/minor allele homozygous = 2.

The final scores were subtracted from the maximum of 1748 (a score of ‘2′ at each of the 874 SNPs). In other words, we determined relatedness by assessing the likelihood of sharing rare (known) genotypes. Orthogonally, we categorized each animal based on their mitochondrial and Y-chromosome haplotypes. We defined families as groups of mice which: (a) shared mitochondrial and Y-chromosome haplotypes, and (b) had a marker score of <500.

### 2.5. Vaccination

Diversity Outbred mice were immunized intramuscularly in the quadriceps, 25 µL per leg, with 5 µg in 50 µL of total PBS volume. The vaccine was composed of the SARS-CoV-2 spike protein and the RIBI Sigma adjuvant system (Sigma), and was prepared according to the manufacturer’s instructions. After 3 weeks of primary vaccination, DO mice again received the same vaccine described above. Three weeks after receiving a boost dose, blood was collected through submandibular bleeds. The collected blood was spun in serum separator tubes to obtain serum.

### 2.6. Virus Challenge

The mouse challenges were conducted under biosafety level 3 conditions (BSL-3) according to standard protocols at the University of North Carolina at Chapel Hill. Mice were sedated with 50 mg/kg of ketamine + 5 mg/kg of xylazine per mouse with Ketamine/Xylazine/PBS, which was administered through intraperitoneal injection. The mice were inoculated with 10,000 pfu of SARS-CoV-2 MA10 virus intranasally in a 50 µL total volume of PBS (Gibco) post-vaccination. Weight measurements were taken on days 0, 1, 2, 3 and 4. The mice were sacrificed on day 4 and the lungs were harvested for viral titer measurement.

### 2.7. Neutralizing Antibody

Serum was obtained from previously vaccinated Diversity Outbred mice against the SARS-CoV-2 spike protein to evaluate antibody neutralizing activity against SARS-CoV-2, as was previously established [32]. The mouse sera was inactivated at 56 °C for 30 min and serially diluted in DMEM supplemented with 10% FBS and 1% pen-strep in a 96-well plate. SARS-CoV-2-nLUC was diluted and approximately 900 PFU/well was added to the diluted sera. The antibody and virus complexes were incubated at 37 °C for an hour. Vero-E6 C1008 cells were maintained in Dulbecco’s modified eagle’s medium (DMEM, Gibco, Life Technologies, Grand Island, NY, USA), 10% fetal bovine serum (FBS, Atlanta Biologicals, R&D Systems, Flowery Branch, GA, USA), and 5% L-glutamine (Gibco, ThermoFisher Scientific, Paisley, United Kingdom). Vero-E6 C1008 cells were plated the previous day in a clear-bottomed, black 96-well plate at a concentration of 2 × 10^4^ cells per well in DMEM with 10% FBS and 1% Pen/Strep and incubated overnight. Once the complexes finished incubating for one hour, they were added to the Vero E6-C1008 cells plated the day before and incubated for 24 h. After 24 h, the Nano-Glo luciferase assay system (Promega) was prepared according to the manufacturer’s recommendations. The cells were lysed, and luciferase activity was measured with the Nano-Glo luciferase assay system. We defined SARS-CoV-2 neutralization at the dilution where a reduction in 50% and 80% of relative light units (RLUs) was observed when compared to the average RLUs present in the virus-positive control wells: 50% or 80% reciprocal inhibitory concentration (IC50, IC80). When correcting for background, an IC50 above 50 indicates positive neutralization activity.

### 2.8. Viral Plaque Assays

The lung viral titers were quantified by plaque assay. The lungs were homogenized in DMEM supplemented with 10% FBS. USAMRIID Vero-E6 cells were plated one day prior to use (2 × 10^5^ cells/well) so as to be 90–95% confluent on the day of the assay. USAMRIID Vero E6 cell monolayers were infected in duplicate with 10-fold serial dilutions of lung homogenates. The plates were rocked every 15 min to ensure the inoculum covered the monolayer evenly. The cells were overlayed with 1.25% carboxymethylcellulose (CMC, Sigma, Saint Louis, MO, USA) and 1× alpha-mem (Corning, Mediatech Inc., Manassas, VA, USA) containing L-glutamine, 5% FBS, 1% HEPES (Gibco Life Technologies, Grand Island, NY, USA), and 1% Penicillin-Streptomycin. The cells were incubated for four days at 37 °C with 5% CO_2_. At 4 days post-infection, infected cell monolayers were fixed overnight with 2% PFA. Post-fixation, the overlay/PFA mixture was removed, and the plates rinsed with water to remove residual CMC. Fixed and washed monolayers were stained with 0.25% crystal violet. Following removal of extra dye and a water rinse, the plaques were counted.

### 2.9. Statistical Analysis

Broad-sense heritability estimates were calculated using Box–Cox transformed values for normality to fit the following linear model:

Phenotype ~ Vaccine Treatment + ε(1)

We then obtained heritability estimates by calculating the following from the linear regression model, where *SS* is the sum of squares of the phenotype being measured:

(*SS_Phen_*)/(*SS_Phen_* + *Residulas*) (2)

A Kolmogorov–Smirnov test was performed for mice in both vaccine categories. The test was performed using the stats package, version 4.1.1 in the R programming software.

Power calculations and correlations were performed using the stats package. Cohen’s coefficient was determined between the PBS and vaccinated groups. Correlations were determined through Pearson’s correlation test.

## 3. Results

### 3.1. Case–Control Design in an Outbred Mouse Population

**Figure 1:**
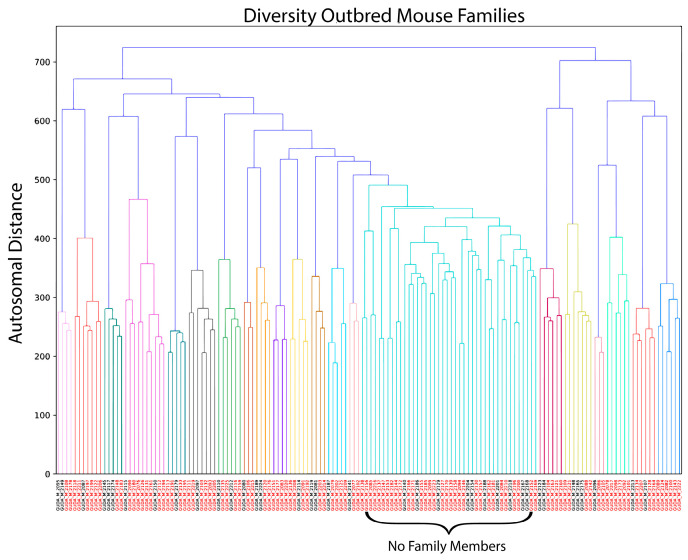
Grouping of Diversity Outbred mice in families. Diversity Outbred mice were separated into different families based on autosomal distance to determine how closely related they are to one another. Each color represents a distinct family of DO mice. The large cluster of green represents mice where no family member could be matched.

To study the effect of genetic variation on SARS-CoV-2 vaccine-induced immunity, we used the Diversity Outbred (DO) mouse population to model genetic diversity within the human population. We obtained 126 male DO mice whose age spanned 10–11 months at the time of vaccination. Based on the sharing of minor alleles (see methods) using the MiniMUGA genotyping array, we determined the relatedness between these animals (Figure 1) [30]. We used this allele sharing, as well as Y- and mitochondrial haplotypes (see methods), to group mice into 35 clusters (hereafter families) with an average of 3 mice/family (range 2–6). In addition, there were 21 mice that could not be grouped with the other mice based on these criteria and were treated as unrelated individuals. The mice were split into two arms: animals were either immunized against SARS-CoV-2 or PBS immunized. Within each family, we assigned members to pairs with the goal of maximizing genetic similarity within each pair (who were then explicitly split between the two immunization arms), across all pairs in a family. Odd-numbered family members and the 21 mice that could not be grouped into families were split evenly between the two immunization arms.

### 3.2. Variable Vaccination and Disease Response across This DO Population

**Figure 2:**
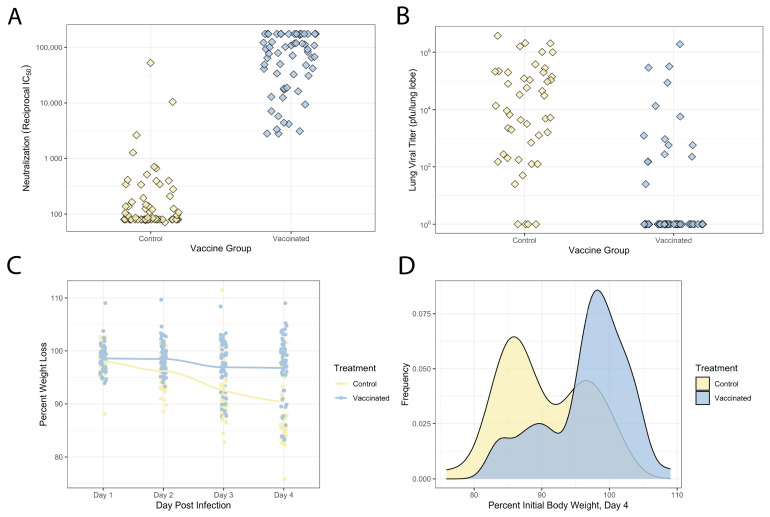
Vaccination response in Diversity Outbred mice is variable, but mostly protective following SARS-CoV-2 challenge. (**A**) Neutralizing antibody titers against MA10 in DO mice from post-boost sera between controls and S2P-vaccinated mice. Neutralization was measured using a virus expressing nanoluciferase and presenting 50% reciprocal inhibitory concentration (IC50). An IC50 above 50 indicates positive neutralization activity. (**B**) Lung viral titers after MA10 challenge in DO mice mock-vaccinated with PBS or spike protein vaccine with Sigma adjuvant (RIBI). Viral titers were measured through plaque assay quantified as PFU/lung lobe. (**C**) Weight loss in PBS mock-vaccinated or spike protein-vaccinated with Sigma adjuvant-RIBI DO mice after MA10 challenge, measured over the course of 4 days. Weight loss percentage was determined based on starting weights prior to infection. (**D**) Distributions of weight loss at day four in DO mice vaccinated with PBS mock vaccination or the spike protein vaccine with Sigma adjuvant-RIBI. Frequency in distributions indicates number of mice for each corresponding weight loss percent. The Kolmogorov–Smirnov test was used to differentiate between control and S2P groups, control: *p* < 2.2^−16^, S2P: *p* < 2.2^−16^.

The DO mice were vaccinated with 5 µg of the SARS-CoV-2 spike protein (S2P) with RIBI adjuvant, or were sham-vaccinated with PBS. All mice were immunized in a prime/boost strategy (with three weeks between injections). To examine correlates of protection in vaccinated DO mice, we measured serum neutralizing antibody titers on day 24 post-boost, prior to virus challenge. Overall, S2P-vaccinated mice exhibited robust, but variable vaccine responses with neutralizing antibody titers ranging from 10^3^ to 10^5^ IC50 (50% reciprocal inhibitory concentration) except for one mouse that had neutralization titers below the limit of detection. The majority of the PBS-immunized mice (63.5%) did not have a detectable neutralizing antibody, but a subset of mice had neutralizing antibody titers, with 23 mice having an IC50 above 10^2^, and 4 of these mice having an IC50 between 10^3^ and 10^4^, likely reflecting variation in nonspecific background, cross-reactive antibody levels (Figure 2A).

Given the variation in neutralizing antibody responses seen in the DO mice, we next tested whether this variation had any impact on protection from SARS-CoV-2 replication or disease. The mice were challenged with 10^4^ plaque-forming units (pfu) of the mouse-adapted strain of SARS-CoV-2 (SARS-CoV-2 MA10) between 1 and 6 weeks post-boost [28,29], and monitored for virus-induced disease and weight loss through 4 days post-infection (DPI, Figure 2C,D), at which point the mice were sacrificed and lungs collected to assess viral loads. Consistent with prior studies with SARS-CoV and SARS-CoV-2 in the related CC and pre-CC populations [33,34,35], unvaccinated DO mice showed significant variation both in susceptibility to virus-induced weight loss, as well as viral replication within the lungs. Sham-vaccinated mice showed weight loss by 4DPI that ranged from severe disease (11–25% weight loss, 24 mice) to mild disease (5–10% weight loss, 5 mice) to no disease (0–5% weight loss, 15 mice). We also observed significant virus-induced mortality in the sham-vaccinated group with 19 mice (33% of the cohort) succumbing to infection prior to the 4DPI endpoint. Viral loads were also variable in the surviving sham-vaccinated group at day 4 post-infection, with titers ranging from 10^1^ pfu (the assay limit of detection) to greater than 10^6^ pfu/lung lobe (Figure 2B), and there was a moderate correlation between weight loss and titer in these animals (r = −0.52, *p* = 0.0003221).

S2P vaccination resulted in significant protection from virus-induced weight loss compared to the sham controls. Across the S2P-immunized mice, we observed no mortality. Additionally, 46 S2P-vaccinated mice showed no disease (0–5% weight loss), 4 mice showed mild weight loss (5–10% weight loss), while only 11 mice had severe disease (11–25% weight loss) at 4DPI (Figure 2C,D). The distributions of weight loss between the sham- and S2P-vaccinated groups were significantly different (Kolmogorov–Smirnov test, D = 1, *p* < 2.2^−16^), showing the robust protection provided by S2P vaccination. Furthermore, in this arm we had 61 mice that reached day 4 for weight loss analysis and 59/61 mice have available titer data. The majority of S2P-vaccinated mice (44/59 mice) did not have viral titers above the limit of detection, with only a subset of 15 mice having viral titers >10^1^ (10^1^–10^6^ pfu/lung lobe, Figure 2D). Additionally, 9 of these 15 mice also experienced severe weight loss, demonstrating that a significant subset of vaccinated animals was susceptible to breakthrough infection (~25%) and/or disease (~15%).

### 3.3. Genetic Diversity Contributes to Vaccination Outcomes

**Figure 3:**
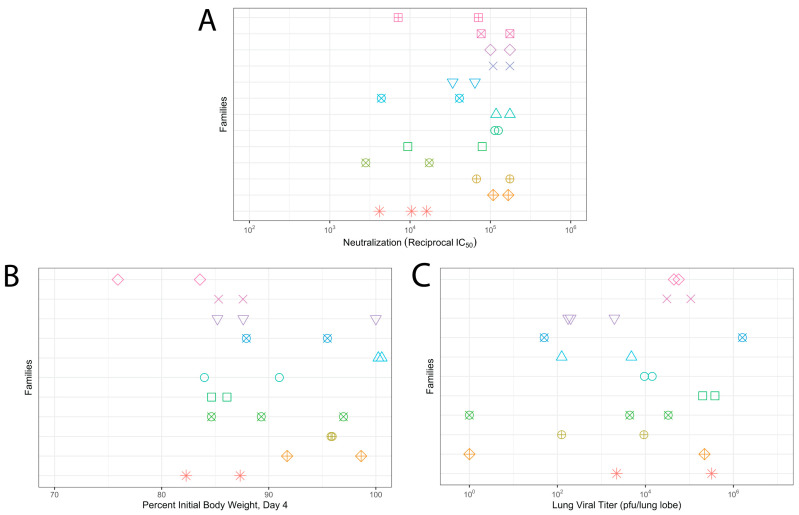
Genetic diversity contributes to vaccination and virus induced disease phenotypes. (**A**) Neutralizing antibody titers measured from serum harvested three weeks post-boost vaccination titers. Neutralization at 50% reciprocal inhibitory concentration (IC50) plotted by family grouping. (**B**) Weight loss across PBS-vaccinated DO mice families measured four days post-MA10 challenge. (**C**) Lung viral titer by family measured from S2P-vaccinated mice challenged with MA10 4 days post-infection. Each color and shape indicates a different family.

To understand the relative genetic contribution amongst DO mice to vaccine-induced neutralizing antibody responses and protection from virus-induced disease, as well as overall susceptibility to SARS-CoV-2-induced disease, we assessed the concordance of each vaccine- or virus-induced phenotype within families. We first analyzed neutralizing antibody titers in the post-boost serum of 29 mice across 13 families, for which we had at least two animals who had received S2P vaccination. The within-family variation in neutralization titers ranged from 0.044 to 0.999 logs IC50 (median 0.357 logs IC50). In contrast, there was up to a 1.33 log difference between some families in IC50 with the median difference between most families being 0.52 logs (Figure 3A). Family was a significant predictor of IC50 titers (*p* = 0.00198). Similar results were found for the more rigorous IC80 titers among the families (Supplementary Figure S1). Given that family was a significant predictor of neutralization titers, we estimated heritability, the percentage of the variance attributed to genetics. We found that family grouping explained approximately 80.6% of the variation in neutralization titers (Table 1), indicating a strong genetic contribution to vaccine success.

Previous studies have identified that responses to SARS and SARS-CoV-2-induced disease are heritable, with genetic variants having been identified that affect disease susceptibility in both mice [22,33,34,35,36], as well as SARS-CoV-2 disease susceptibility in humans [37,38,39,40]. Therefore, we wanted to assess the relative contribution of genetic variation in SARS-CoV-2 disease in the DO, and how this was affected by vaccine status. We therefore analyzed our PBS-immunized mice for both weight loss at 4DPI as well as viral titers in 24 mice across 11 families, for which there were at least 2 mice in a family. Within-family variability in weight loss was between 0.11% and 14.79% (median 6.91%). In contrast, the differences between families spanned from 0.57 to 20.7% weight loss (median 5.56%) at 4 DPI, and family was a significant predictor of weight loss (*p* = 0.04504) (Figure 3B). For the lung viral titer, within-family variability was between 0.11 and 4.81 logs of pfu/lung lobe (median within-family range, 1.87 logs pfu/lung lobe). Differences between families ranged from 0.06 to 2.83 logs (median 1.01 logs pfu/lung lobe), and this difference was not significant (*p* = 0.6793) (Figure 3C). Heritability analysis found that family could explain 68% of the variation in weight loss at day 4 and 41.2% of the variation in lung viral titers (Table 2).

### 3.4. Relationship of Infectious Responses among Vaccine Treatments and Families

**Figure 4:**
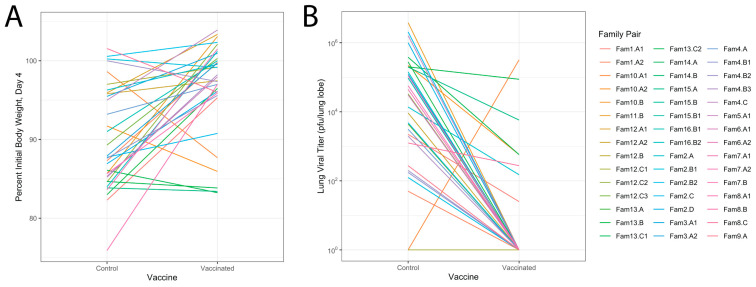
Vaccination is broadly protective in genetically paired mice post-infection. (**A**) Differences in percent weight loss at day 4 between paired mice, based on genetic relatedness, vaccinated with either PBS (control) vaccination or S2P + RIBI. (**B**) Differences in lung viral load measured in pfu/mL between genetic pairs of mice vaccinated with PBS (control) or S2P + RIBI. Pairs were challenged with MA10 at the same time to measure the degree of protection in mice.

We were able to broadly show that, in our population, S2P vaccination strongly skewed the disease responses across this outbred population away from severe disease, and that there was some genetic control of neutralizing antibody titers. However, given the underlying variability in genetic susceptibility to the SARS-CoV-2 disease itself, it is challenging to directly estimate the true protective effects of the vaccine across this outbred and phenotypically variable population. We therefore took advantage of highly related pairs of relatives in our DO population to attempt a better estimation of the protective effects of the vaccine. In total, we identified 46 pairs of mice (across 34 families) which were highly related, infected within the same experimental batch, and were exposed to different vaccine treatments. We calculated the difference in weight loss between the PBS- and S2P-immunized mouse of each pair. Eleven out of these 45 pairs (24%) had the sham-immunized mouse succumb to infection by day 4, while none of the vaccinated mice showed mortality, a highly significant skewing (*p* = 0.005278). For the surviving pairs, PBS-immunized mice in each pair lost on average 10% more weight than the S2P-immunized mouse, although there was considerable range in these weight loss differences (0–22%, Figure 4A). Finally, eight pairs (17%) had the S2P-immunized mouse lose more weight than their sham-immunized partner, ranging from a 1 to 11% weight loss difference.

We conducted a similar analysis on viral loads. Here we could only assess the 34 pairs where both animals survived until harvest. The average reduction in titer was 4 logs, with a range of a 2–4 log reduction between the pairs (Figure 4B). There was one pair where there was no difference in titer and only one pair of mice where lung viral titers were higher in S2P mice than PBS mice, and that difference was 2 logs. All told, our results indicate that immunization with the S2P vaccine is highly protective. However, there do appear to be some genetic backgrounds where efficacy is more muted, and most provocatively, there may be genetic backgrounds where vaccination in fact exacerbates disease outcomes.

## 4. Discussion

While the rapid development and deployment of SARS-CoV-2 vaccines played a major role in reducing morbidity and mortality, there is significant individual-to-individual variation in vaccine responses and this impacts overall population-wide efficacy. This variation in vaccine efficacy is likely driven by a variety of factors. There is a growing body of evidence suggesting that host genetic variation can have a major impact on immune function, including vaccine-induced immunity [12,14,41,42]. Studying the impact of genetic variation on vaccine response and protection is often challenging in humans because it is difficult to control for confounding non-genetic factors, such as prior pathogen exposure, pre-existing immunity, and many other factors influencing immune response. While standard inbred mouse strains have been useful for controlling these environmental factors, these strains do not capture the genetic variation seen in humans. Therefore, we used DO mice to model the genetic variation observed in the human population and understand how genetic variation affects vaccination response outcomes. Our analyses found high levels of variation both in vaccine induced neutralizing antibody responses, and also in vaccine mediated protection from virus-induced disease. Furthermore, our case–control experimental design provided evidence that host genetic factors can modulate vaccine-induced anti-SARS-CoV-2 immunity.

There is growing recognition of the value of mouse genetic reference populations (GRPs), such as the DO or CC, in studying the impact of host genetic diversity on the response to virus-induced disease or vaccination. While standard inbred strains, such as BALB/cJ or C57BL/6J mice, have long been used to test the safety and efficacy of experimental vaccines, including SARS-CoV-2 vaccines, these strains do not accurately model the genetic and phenotypic diversity seen in human populations [20,43,44,45]. Importantly, all the SARS-CoV-2 vaccines currently in use within humans show variation in their efficacy within human populations, even against homologous virus challenge [46,47,48,49,50]. However, early preclinical studies with these vaccines using inbred mouse strains generally showed uniform immunogenicity and protection against SARS-CoV-2 infection [18,44,51,52,53]. Although variation is observed in lower vaccine doses (<1 µg), mouse studies analyzing neutralizing response in inbred strains with protein vaccination and similar adjuvants have little variation in neutralizing antibody titers [54,55,56].Therefore, one of our goals was to determine whether the DO mice would more accurately reflect the variation in vaccine immunogenicity and efficacy seen in human populations. This was borne out in our studies, where the DO mice showed over 100-fold variation in the magnitude of vaccine-induced neutralizing antibody responses. Furthermore, we observed a significant fraction of vaccinated mice showing signs of vaccine breakthrough as indicated by 11 mice with severe virus-induced weight loss, 15 mice with residual viral titers in the lungs, and 9/59 mice with both. This analysis suggests that host genetic variation impacts SARS-CoV2 vaccine performance, and that DO mice, or related populations such as the CC, could be used as a complement to existing inbred mouse models to evaluate the impact of host genetic variation on vaccine efficacy or safety before moving on to testing in more advanced models such as ferrets or non-human primates.

Several human GWAS studies have identified host genes and genetic loci associated with variation in SARS-CoV-2 disease severity and long-COVID [37,38,39,57,58,59,60,61,62]. Analysis of the impact of host genetic variation on SARS-CoV-2 vaccine-induced immunity has found associations between variant HLA alleles and variation in vaccine-induced antibody responses, vaccine breakthrough and vaccine side effects [63,64]. Small-scale pilot studies have also identified loci associated with variation in antibody responses following vaccination with an inactivated vaccine [65]. Our results, showing a significantly heritable response to both vaccine efficacy and reduced disease post challenge, suggest that the DO and related mouse populations can be used to complement human studies to understand the role of genetic variation in regulating vaccine responses. DO mice can also serve as a valuable resource in future work to understand the variation across vaccine platforms in humans and variation specific to protein subunit vaccines. It would further help untangle whether variation in immune response is due to the vaccine antigen or adjuvants. To our knowledge, this is the first time that DO mice have been used to study the contribution of host genetic variation to SARS-CoV-2 vaccine-induced immunity. Further, prior studies with CC mice have shown good concordance with human GWAS studies that identified genetic regulators of severe SARS-CoV-2-induced disease suggesting further work in assessing host genetic drivers of vaccine efficacy will be translatable between mouse and human [36,66].

Neutralizing antibody levels are known to be a reliable and robust correlate of protection for human COVID-19 vaccine efficacy [67,68]. However, a strong neutralizing antibody response may not always prevent breakthrough disease from occurring. Susceptibility loci to SARS-CoV and SARS-CoV-2 have been identified in CC mice and can impair immune function and worsen disease phenotypes [34,36]. These susceptibility loci can impact vaccine-induced immune cell response and result in poor vaccine performance. This potentially allows breakthrough infections to occur as a robust antibody response may not overcome effects of susceptibility loci. Similarly, human studies with COVID-19 have shown vaccinated individuals produced robust neutralizing antibody to SARS-CoV-2 and VOCs prior to infection, but still experienced breakthrough disease [69,70,71,72]. Therefore, relying on neutralizing antibody alone may not be the best way to assess potential vaccine protection since it has long been recognized that correlates of protection are multi-faceted, especially across large populations. Consistent with these observations, in this study, despite robust neutralizing titer levels in our DO mice, a significant portion of mice showed mild to severe weight loss (25%) and/or viral titers (30%). In addition, recent work on mycobacterium tuberculosis vaccination showed that despite robust protection across various CC strains, there were no common correlates of protection in this study [73]. Taken together, such results suggest that outbred populations, such as the DO, may be critical for modeling more nuanced protective features of vaccines.

As discussed above, the DO mice and related resources represent powerful tools for understanding the impact of host genetic variation on a wide range of host responses, and these systems could be used to identify the specific polymorphic host genes associated with this variation. However, there are a number of other important questions in coronavirus vaccinology, and vaccinology in general, where these types of resources might be useful. While our analysis focused on neutralizing antibody responses, there is a growing body of evidence suggesting that T cells and/or non-neutralizing, virus specific antibodies may contribute to heterologous vaccine-mediated protection [74,75]. It will be important to evaluate whether host genetic variation contributes to differences in these responses and how that affects protection against heterologous coronavirus challenge. Furthermore, previous human studies have found that disease severity and inflammatory cytokine profiles are lower in vaccinated than unvaccinated individuals, but are influenced by various risk factors [76,77]. While SNPs have been identified in association with lower inflammatory cytokine levels in vaccinated populations affecting vaccine protection, the impact of genetic variation in disease severity after vaccination remains poorly defined [78]. Therefore, the DO may be a particularly useful resource for modeling how host genetic variation affects these responses in vaccinated or unvaccinated individuals. The variation in vaccine efficacy seen in the DO mice also creates an opportunity to use this resource as a platform for testing different adjuvants or adjuvant systems for their ability to overcome vaccine failure and elicit protection even in individuals or family groups that are genetically predisposed to vaccine failure.

Understanding the specific polymorphic host genetic factors that drive the variation in vaccine efficacy would enhance our ability to develop vaccination strategies that are more effective. While our group sizes were not sufficiently powered to perform genetic mapping studies, these results provide the support necessary to conduct large-scale studies on DO mice or the related CC population. With a large-scale mapping study, it would be possible to identify SARS-CoV-2 susceptibility loci that influence vaccination efficacy across the population. Our work suggests future studies would require a case–control design to detect loci directly impacting vaccine efficacy and virus induced disease, while avoiding confounding underlying susceptibility loci. The size of such an experiment would depend on many factors, including the genetic architecture of both the primary infection and vaccine responses such as neutralizing antibody, viral load, and virus-induced weight loss. Our data, and assumptions of one or a few loci of moderately large effect, suggest that such a study would require approximately 420 DO animals in each vaccine group to identify a locus for weight loss in mice. However, approximately 120 mice in each group would be necessary to detect a locus for neutralizing antibody or viral load. Although, we note that there are a variety of other mapping populations that could be generated to address these questions [33,79].

Our results demonstrate that DO mice can be used to reproduce the variation in vaccine outcomes seen in human populations, and that heritable genetic variation plays a major role in determining vaccine efficacy. Vaccines are designed to protect as many individuals as possible from developing disease symptoms or severe disease, yet existing preclinical model systems do not account for the impact of host genetic variation on vaccine immunogenicity or efficacy. Therefore, the use of preclinical models such as the DO would allow investigators to rapidly and inexpensively test vaccine performance in the face of host genetic diversity prior to moving into more advanced models. Furthermore, understanding the specific polymorphic host genes/pathways that affect vaccine performance may inform the development of vaccines and/or adjuvant combinations that are capable of eliciting robust responses across genetic backgrounds. Therefore, using genetically complex mouse populations in combination with human populations to identify the role of polymorphic host genes in regulating vaccine-induced immunity may provide new avenues for improving vaccine performance in humans.

## Figures and Tables

**Table 1:** Phenotypic heritability of neutralizing antibody titers for S2P-vaccinated DO families.

Treatment	IC50 Heritability	IC80 Heritability
Vaccinated	80.6%	74.3%

**Table 2:** Phenotypic heritability of day 4 weight loss and lung viral titer for PBS-vaccinated DO families.

Treatment	Day 4 Percent Weight Loss Heritability	Vaccinated Lung Viral Titer Heritability
PBS	68.0%	41.2%

## Data Availability

The data presented in this study are available within the document and Supplementary File.

## Supplementary Materials

The following supporting information can be downloaded at: https://www.mdpi.com/article/10.3390/vaccines12010103/s1, Figure S1: IC80 Neutralizing Antibody Titers; Data S1: DO Sorted Autosomes; Data S2: DO MT_Y sorted.
