# Supplementary material for: Host Genetic Variation Impacts SARS-CoV-2 Vaccination Response in the Diversity Outbred Mouse Population
Source: Vaccines (Basel). 2024 Jan 20;12(1):103. doi: 10.3390/vaccines12010103 (PMC10821422; doi:10.3390/vaccines12010103)
Supplement: Supplementary file 1 [file vaccines-12-00103-s001.zip › Supplementary Figure S1_DO.pdf]

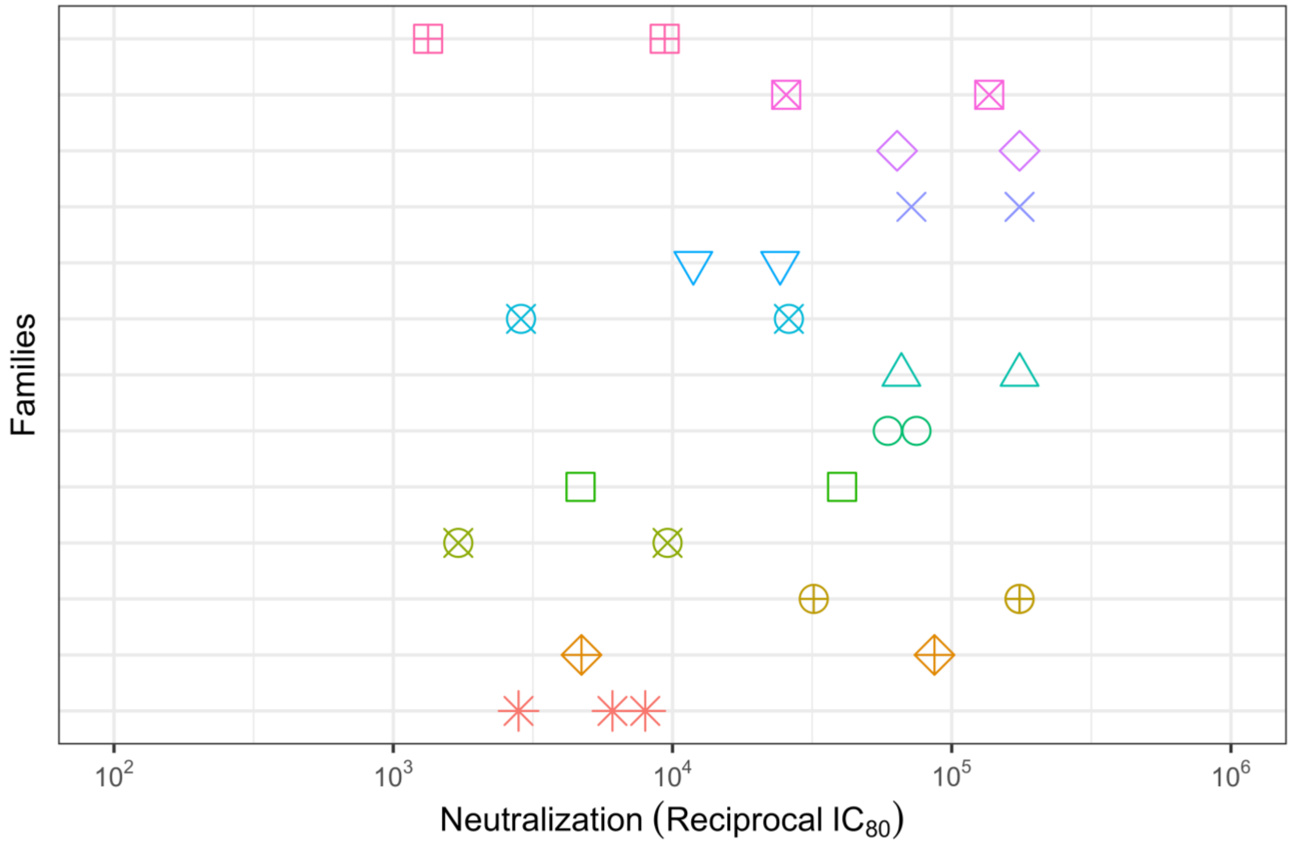

Supplementary Figure S1. IC80 neutralizing antibody titers measured from serum harvested three weeks post boost vaccination titers.
